# Supplementary material for: Conformational antigenic heterogeneity as a cause of the persistent fraction in HIV-1 neutralization
Source: Retrovirology. 2023 May 27;20:9. doi: 10.1186/s12977-023-00624-9 (PMC10221750; doi:10.1186/s12977-023-00624-9)
Supplement: Supplementary file 1 — Additional file 1: Table S1. Validation of Langmuir modelinga. Table S2. Validation of heterogeneous-ligand modelinga. Figure S1. Analysis of bNAb binding to differentially bNAb-purified B41 SOSIP.664 by ELISA. Each diagram shows the binding of one bNAb to BG505or B41SOSIP.664 trimers purified in three ways. The optical densitiesare plotted on the y axes as functions of the bNAb concentrations. Figure S2. Extent of neutralization in vitro as a predictor of protection in vivo. [file 12977_2023_624_MOESM1_ESM.pdf]

Additional File - Colin et al. Supplementary Tables and Figures

Supplementary information

SI Table 1. Validation of Langmuir modeling [a]

SOSIP.664 trimer	Fab	Affinity purification	*T* (k_{on})[b]	*T* (k_{off})[b]	$\chi^{2\,b}$	k_t[b] (RU/Ms)	*T* (k_t)[c]
**BG505**	PGT145	2G12 (n=3)	$9.4 \cdot 10^2$ ± 33	$1.2 \cdot 10^3$ ± 67	$4.4 \cdot 10^{-2}$ ± $5.9 \cdot 10^{-3}$	$1.0 \cdot 10^{15}$ ± $7.0 \cdot 10^{14}$	1.5 ± 0
	PGT151	2G12 (n=2)	$3.0 \cdot 10^2$ ± 5.0	$1.3 \cdot 10^2$ ± 0	0.62 ± $1.8 \cdot 10^{-2}$	$2.0 \cdot 10^{22}$ ± $1.3 \cdot 10^{22}$	1.5 ± 0
**B41**	PGT145	2G12 (n=3)	$1.6 \cdot 10^2$ ± 3.3	51 ± 2.9	0.12 ± 0	$2.5 \cdot 10^{16}$ ± $1.4 \cdot 10^{16}$	0.14 ± $7.3 \cdot 10^{-2}$
		PGT145 (n=3)	$2.1 \cdot 10^2$ ± 18	3.2 ± 3.1	0.19 ± $2.2 \cdot 10^{-2}$	$4.8 \cdot 10^9$ ± $2.4 \cdot 10^9$	1.5 ± 0.46
	PGT151	2G12 (n=3)	$1.2 \cdot 10^2$ ± 16	$1.4 \cdot 10^2$ ± 18	0.13 ± 3.5 . 10^{-2}	$1.2 \cdot 10^{22}$ ± $1.2 \cdot 10^{22}$	$6.3 \cdot 10^{-4}$ ± $3.1 \cdot 10^{-4}$

[a] Tabulated values are means ± S.E.M of *n* independent replicates.
[b] Kinetic parameters were validated by global fitting. *T* value > 10 confirms the statistical significance of the parameter value. χ^2 values, the average squared residuals between experimental and fitted data, were determined as measures the goodness of the fit.
[c] The mass transfer constant = $k_t > 10^9$ and low value (<10) of T(*kt*) indicate lack of significant mass-transfer limitation.

SI Table 2. Validation of heterogeneous-ligand modeling [a]

SOSIP.664 trimer	Fab	Affinity purification	*T* (k_{on1})[b]	*T* (k_{off1})[b]	*T* (k_{on2})[b]	*T* (k_{off2})[b]	$\chi^{2\,b}$
**BG505**	PGT145	2G12 (n=3)	$9.8 \cdot 10^2$ ± 61	0.47 ± 0.10	$1.3 \cdot 10^2$ ± 6.7	$3.6 \cdot 10^2$ ± 27	$1.5 \cdot 10^{-2}$ ± $1.6 \cdot 10^{-3}$
	PGT151	2G12 (n=2)	$2.5 \cdot 10^2$ ± 10	0.15 ± $5.0 \cdot 10^{-3}$	93 ± 0.50	78 ± 1.5	0.10 ± $2.0 \cdot 10^{-3}$
**B41**	PGT145	2G12 (n=3)	83 ± 26	44 ± 11	$1.8 \cdot 10^2$ ± 22	39 ± 8.0	$8.0 \cdot 10^{-2}$ ± $1.1 \cdot 10^{-2}$
		PGT145 (n=3)	$1.1 \cdot 10^2$ ± 45	27 ± 10	$1.9 \cdot 10^2$ ± 29	25 ± 3.5	0.13 ± $2.7 \cdot 10^{-2}$
	PGT151	2G12 (n=2)	$1.1 \cdot 10^2$ ± 11	$1.3 \cdot 10^2$ ± 47	95 ± 8.0	$1.2 \cdot 10^2$ ± 25	0.10 ± $1.2 \cdot 10^{-2}$

[a] Tabulated values are means ± S.E.M of *n* independent replicates.
[b] Kinetic parameters were validated by global fitting. *T* value > 10 confirms the statistical significance of the parameter value. χ^2 values, the average squared residuals between experimental and fitted data, were determined as measures the goodness of the fit.

SI Figures

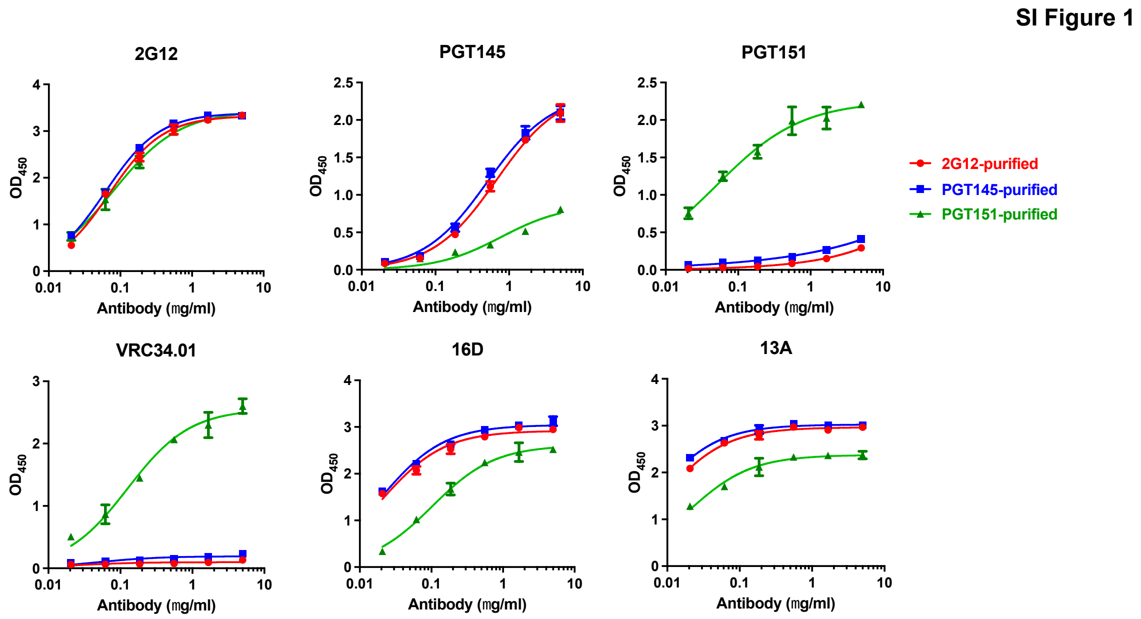

SI Figure 1. Analysis of bNAb binding to differentially bNAb-purified B41 SOSIP.664 by ELISA. Each diagram shows the binding of one bNAb to BG505 (top row) or B41 (bottom row) SOSIP.664 trimers purified in three ways (color-coded legend). The optical densities (OD_{450}) are plotted on the y axes as functions of the bNAb concentrations.

SI Figure 2

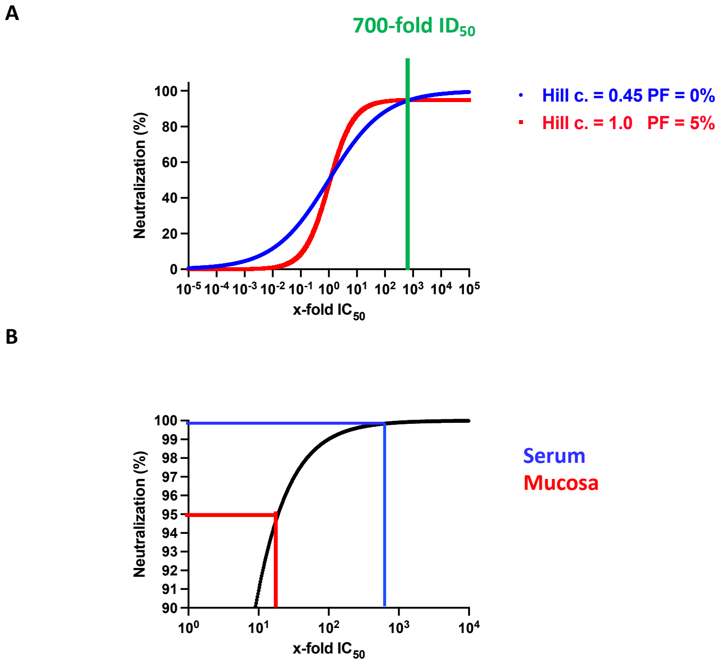

SI Figure 2. Extent of neutralization *in vitro* as a predictor of protection *in vivo*. Extent of neutralization is depicted on the y axis (%) as a function the x-fold excess of NAb concentration over IC_{50} in the neutralization reaction (mixture of NAb and virus). **A.** The diagram elaborates on a meta-analysis of data from passive immunization of macaques with bNAbs followed by SHIV challenge [1]. Logistic modeling showed that protection of 95% of animals required an ID_{50} of the animal sera at challenge of ~700 (green vertical line). The meta-analysis suggested that 50% protection *in vivo* corresponded to 93.7% neutralization *in vitro*, 75% protection to 99.8% neutralization, and 95% protection to > 99.9% neutralization. That was under the assumption of an asymptotic approach to 100% neutralization, but the values for instantaneous inhibitory potential were based on the empirical data. The red and blue curves illustrate possibilities for smaller discrepancies between protection *in vivo* and neutralization *in vitro*. The arbitrary coincidence of 95% protection and neutralization is chosen as an example. NAbs present at 700-fold higher concentration than their IC_{50} (*i.e.*, ID_{50} of the solution is 700) would neutralize 95% of the virus input, given the Hill coefficient = 0.45 and PF = 0% or a Hill coefficient = 1.0 and PF = 5%. Under the assumption of similar ID_{50} (a product of NAb affinity and concentration [2]) and efficacy, *in vivo* and *in vitro*, the example might be deemed to show realistic correspondences. The unknown is the degree of neutralization of the inoculum *in vivo* in the relevant compartment, *i.e.*,

perhaps exclusively at the portal of entry. In **B** the condition that the ID_{50} is the same *in vitro* and *in vivo* is relaxed. Neutralization *in vitro* is mediated by NAbs in serum; protective neutralization *in vivo* is postulated to occur at the mucosal site of viral deposition. The concentration of the infused NAb is known to be lower in the mucosal interstitial fluid and lumen than in the sera, and more markedly so intrarectally than intravaginally [3], reducing ID_{50} accordingly. Furthermore, virus and target cells differ in important respects between *in vivo* and *in vitro* conditions, which may also reduce the ID_{50} [4-7]. The diagram shows a simulation of the degree of neutralization under the realistic assumption that ID_{50} is 37-fold lower in the mucosa than in serum through the combined NAb concentration and potency reductions, *e.g.*, 3.7-fold of the former and 10-fold of the latter. The serum ID_{50} value of 700 from [1], corresponding to protection of 95% of the animals, would, with asymptote 100% and Hill coefficient 1.0, give 99.86% neutralization, which might seem high considering the proportion of infected animals. But with the above assumptions about the mucosa, neutralization there would be 95%. We are not arguing that percentages of neutralization in the mucosa and protection of animals have to be identical, just that under realistic assumptions they do not necessarily differ much.

REFERENCES

1. Pegu A, Borate B, Huang Y, Pauthner MG, Hessell AJ, Julg B, Doria-Rose NA, Schmidt SD, Carpp LN, Cully MD, et al: **A Meta-analysis of Passive Immunization Studies Shows that Serum-Neutralizing Antibody Titer Associates with Protection against SHIV Challenge.** *Cell Host Microbe* 2019, **26:**336-346 e333.
2. Klasse PJ: **Neutralization of Virus Infectivity by Antibodies: Old Problems in New Perspectives.** *Adv Biol* 2014, **2014**.
3. Klein K, Veazey RS, Warrier R, Hraber P, Doyle-Meyers LA, Buffa V, Liao HX, Haynes BF, Shaw GM, Shattock RJ: **Neutralizing IgG at the portal of infection mediates protection against vaginal simian/human immunodeficiency virus challenge.** *J Virol* 2013, **87:**11604-11616.
4. Cohen YZ, Lorenzi JCC, Seaman MS, Nogueira L, Schoofs T, Krassnig L, Butler A, Millard K, Fitzsimons T, Daniell X, et al: **Neutralizing Activity of Broadly Neutralizing Anti-HIV-1 Antibodies against Clade B Clinical Isolates Produced in Peripheral Blood Mononuclear Cells.** *J Virol* 2018, **92**.
5. Sarzotti-Kelsoe M, Bailer RT, Turk E, Lin CL, Bilska M, Greene KM, Gao H, Todd CA, Ozaki DA, Seaman MS, et al: **Optimization and validation of the TZM-bl assay for standardized assessments of neutralizing antibodies against HIV-1.** *J Immunol Methods* 2014, **409:**131-146.

6. Sarzotti-Kelsoe M, Daniell X, Todd CA, Bilska M, Martelli A, LaBranche C, Perez LG, Ochsenbauer C, Kappes JC, Rountree W, et al: **Optimization and validation of a neutralizing antibody assay for HIV-1 in A3R5 cells.** *J Immunol Methods* 2014, **409:**147-160.
7. Klasse PJ: **Modeling how many envelope glycoprotein trimers per virion participate in human immunodeficiency virus infectivity and its neutralization by antibody.** *Virology* 2007, **369:**245-262.
